# Weighed Down by Paperwork: Identifying Barriers to Metabolic and Bariatric Surgery Evaluation in the United States

**DOI:** 10.1007/s11695-026-08888-8

**Published:** 2026-08-29

**Authors:** Rylee Barber, Stephen Moorhead, Kalina Siehl, Ronit Pathak, Mary K Bryant

**Affiliations:** 1 Medical University of South Carolina, Charleston, United States; 2 College of Charleston, Charleston, United States

**Keywords:** Barriers to care, Metabolic bariatric surgery, Patient navigation, Obesity

## Abstract

**Background:**

Obesity affects over 42% of U.S. adults, yet access to metabolic and bariatric surgery (MBS) remains limited despite its proven efficacy. While use of GLP-1 receptor agonists has risen in the past three years, MBS volume has declined over 25% during the same period. Reported barriers to MBS include long wait times, societal sigma, fear of surgery, and insurance restrictions. This study investigated appointment accessibility and program requirements for patients seeking MBS.

**Methods:**

We conducted a secret shopper study of bariatric surgery centers in North Carolina (n=18) and South Carolina (n=17) June - August 2025. Trained callers contacted each program twice, once posing as a patient with Medicaid and once as a patient with private insurance, to request an initial consultation appointment. Data collected included appointment wait times, required documentation, pre-surgical prerequisites, and administrative barriers.

**Results:**

Of the 24 centers successfully contacted, all accepted private insurance and 94.3% accepted Medicaid. Median wait time to appointment was 20 days, with no difference by insurance type. On average, programs required 1.7 calls before reaching a scheduler. Most centers (77.1%,n=27) required pre-appointment steps such as an online application, seminar, or PCP referral. Eleven centers could not be reached due to incorrect contact information, and two offered only online intake forms.

**Conclusions:**

Rather than insurance-related disparities, we found that administrative barriers such as repeated call attempts, inaccurate contact information, and variable prerequisites were the main obstacles to MBS access. The lack of standardization across programs highlights the need to streamline the evaluation process.

## Key Points

• Administrative and scheduling barriers may limit access to initial MBS appointments.

• Many centers require pre-visit tasks that add complexity before care begins.

• Contact information issues make some centers difficult to reach.

• Scheduling often requires multiple attempts, with wait times of several weeks.

## Introduction

Obesity is a significant public health crisis because of its high prevalence, associated morbidity and mortality, and substantial economic burden [1]. Despite this, metabolic and bariatric surgery (MBS), the most effective treatment for achieving sustained weight loss, is utilized by only 1–2% of eligible patients [2]. Research has previously shown that attendance to the initial MBS appointment results in higher rates to completion of surgery [3]. Studies are lacking in appointment accessibility, and potential barriers to attendance. This study investigated appointment accessibility and program requirements for patients seeking MBS in two states of the USA.  

## Methods

**Fig. 1:**
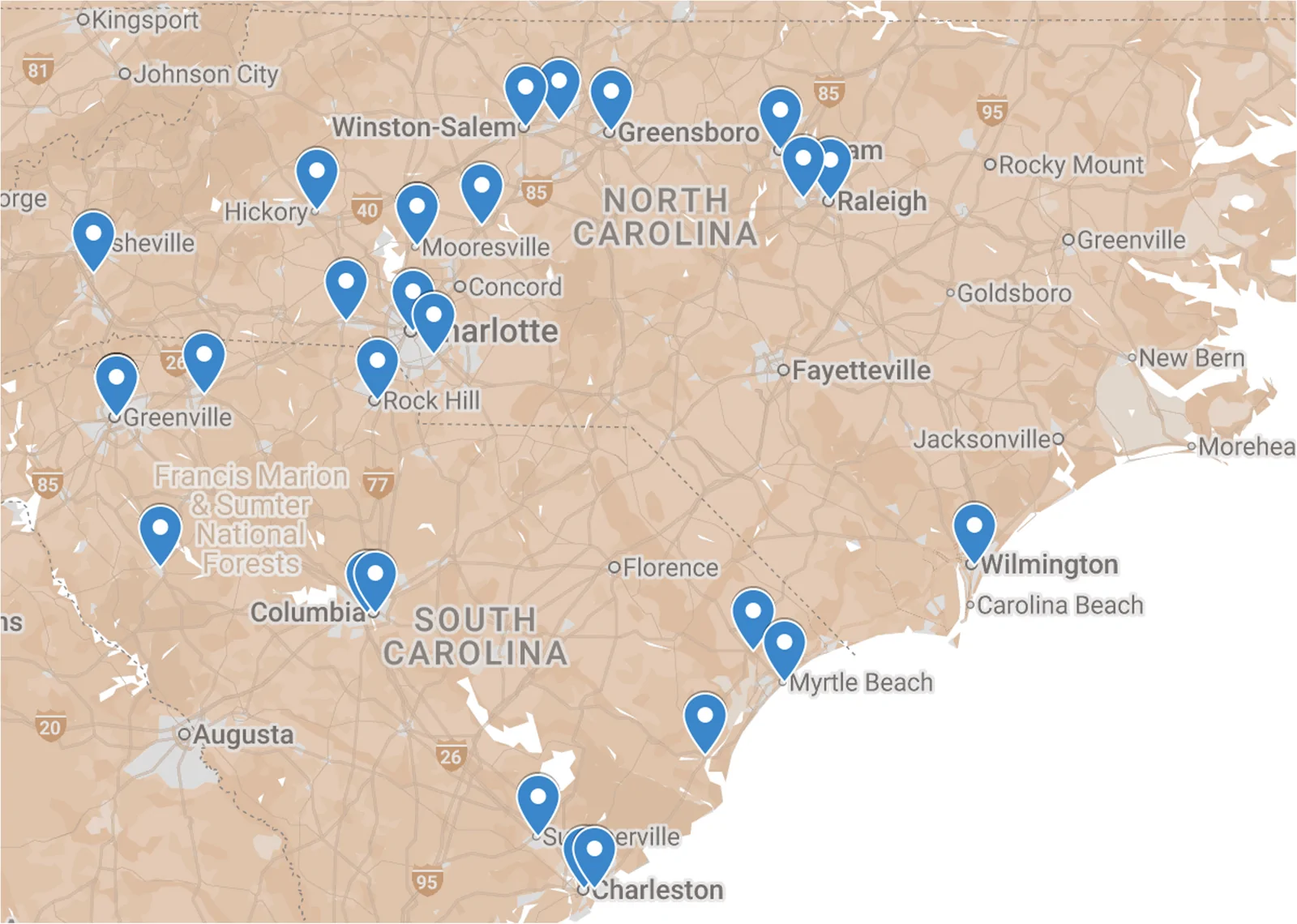
Map of MBS programs in North Carolina and South Carolina

We conducted a secret shopper study of metabolic and bariatric surgery centers in North Carolina (*n* = 18) and South Carolina (*n* = 17) June - August 2025 locations shown in Fig. 1. Metabolic and bariatric programs were obtained from publicly available sources. A random-sequence generator (Random.org, Dublin, Ireland) [4] was used to randomize the order in which centers were called within each set. Trained callers contacted each program twice, once posing as a patient with Medicaid and once as a patient with private insurance. The order in which the insurance scenario each practice received was also randomized. The callers explicitly confirmed insurance acceptance during the calls. If during the call, a practice accepted the insurance type but required insurance number or government information for an appointment date, the callers did not provide any falsified information. Calls were conducted between 9 − 3 pm EST, to request an initial consultation appointment. Repeat calls to each practice were separated by at least 2 weeks. Callers were provided scripted responses, including demographics and clinical details, as needed for the appointment screening process. Calls that did not obtain an appointment were excluded from the analysis for appointment wait time. Data collected included appointment wait times, required documentation, pre-surgical prerequisites, and administrative barriers. If programs had incorrect or nonfunctional contact information or could not be reached after three attempts on different days, this was documented as access barrier.

## Results

**Fig. 2:**
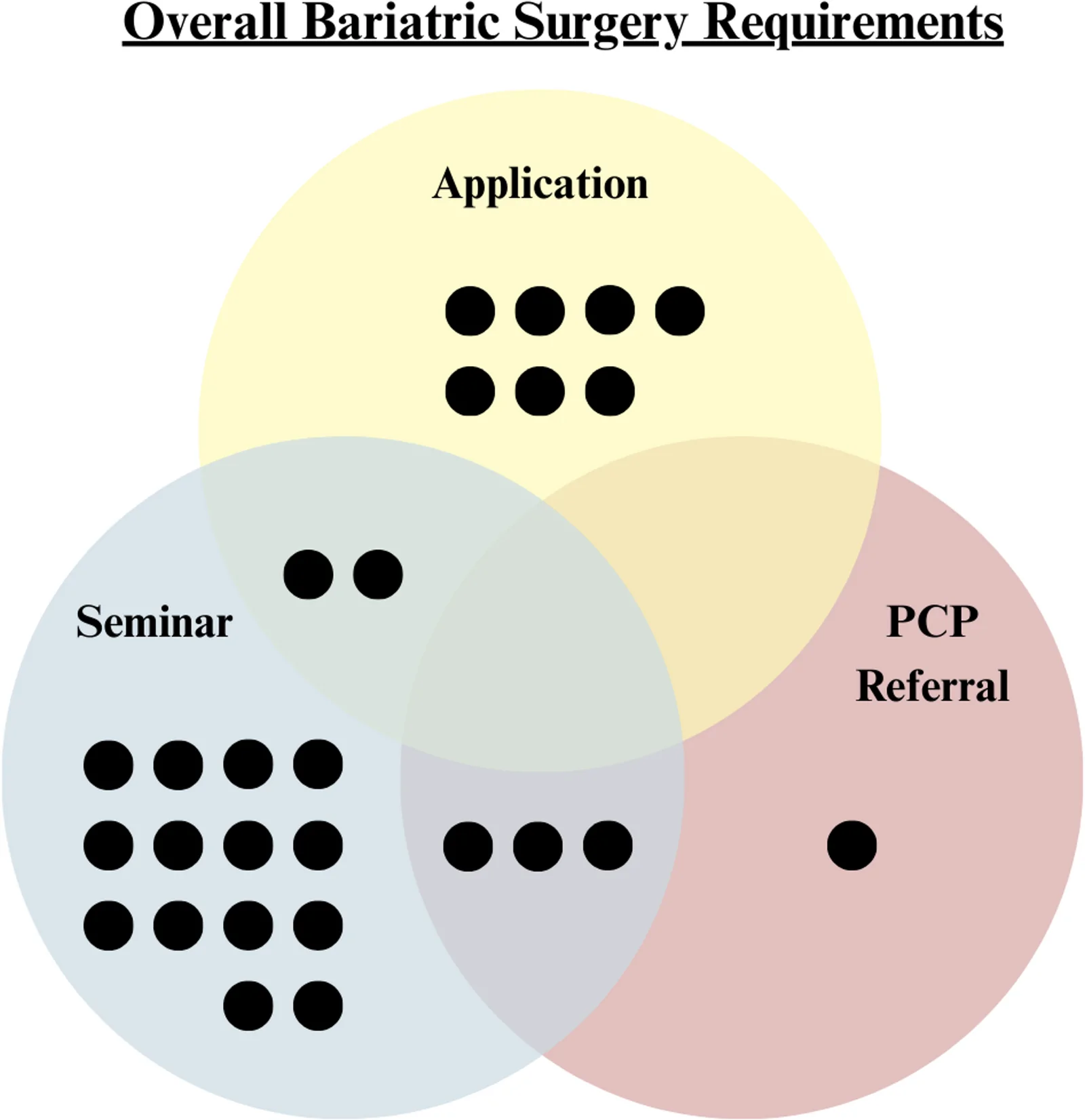
Pre-appointment requirements for metabolic and bariatric surgery programs in North Carolina and South Carolina

Between South Carolina and North Carolina, 24 of 35 (68.6%) centers were successfully contacted by telephone. Of centers with successful telephone encounters, all accepted private insurance and 94.3% accepted Medicaid. Median wait time to appointment was 20 days [3–62], with no difference by insurance type. On average, programs required 1.7 [1–4] calls before reaching a scheduler. Most centers (77.1%, *n* = 27) required pre-appointment steps: online application (45.8%, *n* = 16), seminar attendance (29.2%, *n* = 10), PCP referral (25%, *n* = 9) and screening questionnaires (20.8%, *n* = 7) (Fig. 2**)**. Of the 24 centers, 8 (22.9%) required completed two or more steps before appointment could be scheduled. Eleven centers (31.4%) could not be reached despite multiple contact attempts. Nine centers had incorrect contact information and two only offered online intake forms.

## Conclusion

This study found that rather than insurance-related disparities, administrative barriers such as repeated call attempts, inaccurate contact information, and variable prerequisites were the identified obstacles to MBS appointment access in North and South Carolina. Despite multiple contact attempts, 31.4% of centers could not be reached, representing a significant and potentially modifiable barrier to access for MBS. In the setting of an obesity epidemic, these readily correctable barriers to care must be mitigated for patients’ health. On average, 1.7 calls were required to reach a scheduler, which may pose a barrier for patients with limited time. Most centers required pre-appointment steps (77.1%), and nearly one quarter (22.9%) required multiple steps before an appointment could be scheduled, which may disproportionately burden patients with limited time, lower health literacy, or reduced social support. The median wait time for an initial appointment was 20 days, which prolongs the time to metabolic and bariatric surgery and delays the benefits of MBS. Longer wait times in other fields are also associated with higher no-show rates [5]. Pitzul et al. found in their cohort of patients that when patients were assessed by a health-care professional only 13.2% did not continue to MBS treatment [3]. This is an extremely low rate and may be more site specific; however, it does instill the importance of streamlining the process of patients obtaining their initial appointment. Obesity is a significant health crisis that can be effectively reversed with MBS; however, this study found that simple administrative tasks stand between many patients and this procedure. Metabolic and bariatric surgery centers should work to streamline the process of getting patients into their clinics, and analyze how their practice may be creating barriers to care for these patients. Patients face enough barriers without their own healthcare providers creating more.

## Data Availability

No datasets were generated or analysed during the current study.
